# Supplementary material for: Molecular Action Mechanism of Coixol from Soft-Shelled Adlay on Tyrosinase: The Future of Cosmetics
Source: Molecules. 2022 Jul 20;27(14):4626. doi: 10.3390/molecules27144626 (PMC9325028; doi:10.3390/molecules27144626)
Supplement: Supplementary file 1 [file molecules-27-04626-s001.zip › molecules-1790705-supplementary.pdf]

Supplementary

**One-OH polyphenolics occurring in Coicis**	
Coniferyl alcohol	Syringic acid,
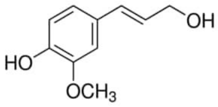	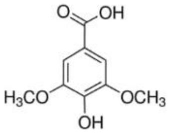
trans-coniferylaldehyde	Sinapaldehyde,
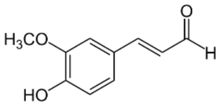	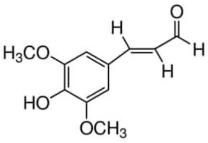
Ferulic acid	Syringaldehyde
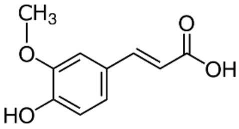	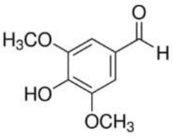
p-Hydroxybenzaldehyde	Vanillin,
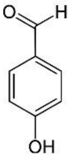	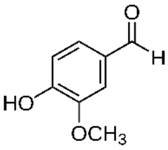
**Two-OH polyphenolics/flavonoids occurring in Coicis**	
Caffeic acid	Syringaresinol
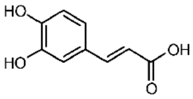 Caffeic Acid	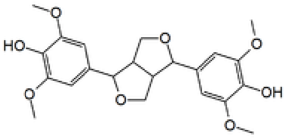
Chlorogenic acid	4-ketopinoresinol
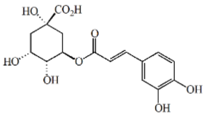	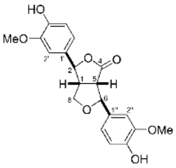
Naringin	
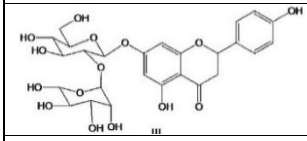	
**Three-OH polyphenolics/flavonoids occurring in Coicis**	
Gallic acid	Naringenin

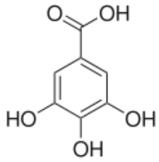	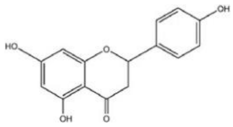
**Others**	
Coixenolide, (11E)-11-Octadecenoic acid (1S,2R)-1-methyl-2-[[(9Z)-1-oxo-9-hexadecenyl]oxy]propyl ester	Mayuenolide, a lignan
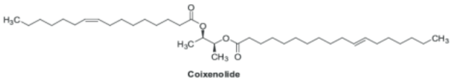	
Ceramide	
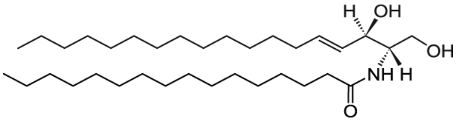	

Figure S1. The cited main polyphenolics and flavonoids identified in coicis.*.
*Rearranged from Kuo *et al*.(2012) and Ragasa et al., (2014) [4,23].

**Heterocyclic compounds occurring in Coicis**	
Coixspirolactams A-E	
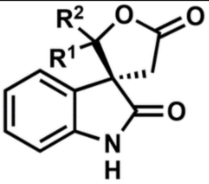<br>**Coixspirolactam A**, $R^1 = R^2 = H$<br>**Coixspirolactam B**, R^1 = Me, R^2 = H; **Coixspirolactam C**, R^1 = H, R^2 = Me<br>**Coixspirolactam D**, R^1 = Ac, R^2 = H; **Coixspirolactam E**, R^1 = H, R^2 = Ac	
Methyl dioxindole-3-acetate	Coixol (6-methoxy-2-benzoxazolinone)
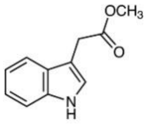	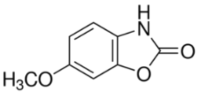

Figure S2. Main heterocyclic compounds identified in coicis.*.
*Rearranged from Kuo *et al*.(2012) and Ragasa et al., (2014) [4,23].

Potent anti-hTyrosinase compound	Potent anti-fruit tyrosinase compound
Thiamidol (isobutylamido-thiazolyl-resorcinol)	Omeprazole **(5-Methoxy-2-{[(4-methoxy-3,5-dimethyl-2-pyridinyl)methyl]sulfinyl}-1H-benzimidazol)**
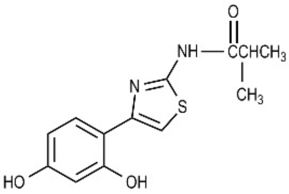	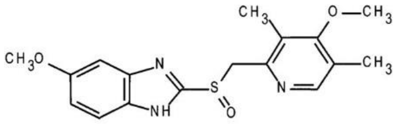

Figure S3. Potent antityrosinase compounds.

a) Taichung No. 1

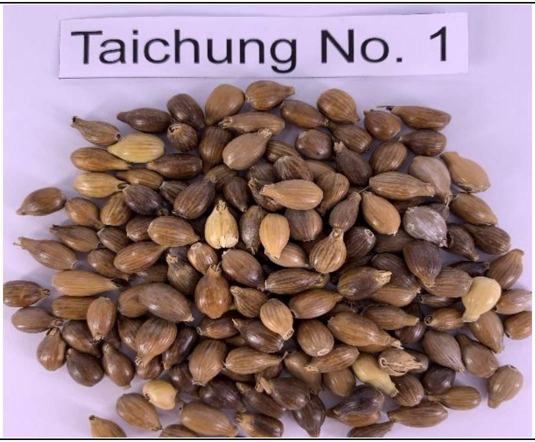

b) Taichung No. 2

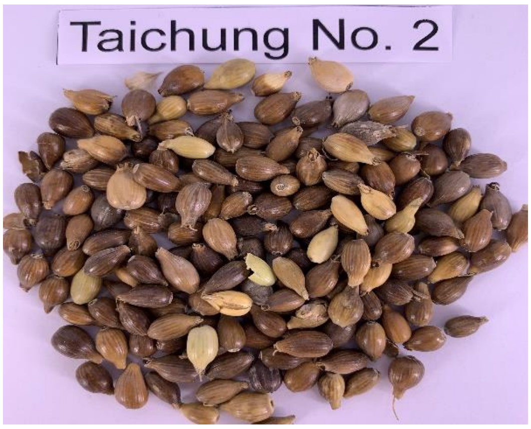

c) Taichung No. 3

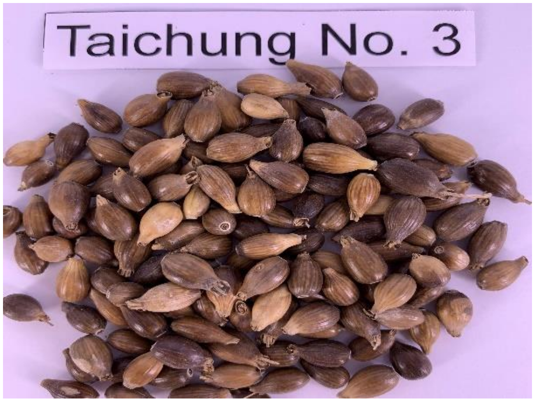

d) Taichung No. 4

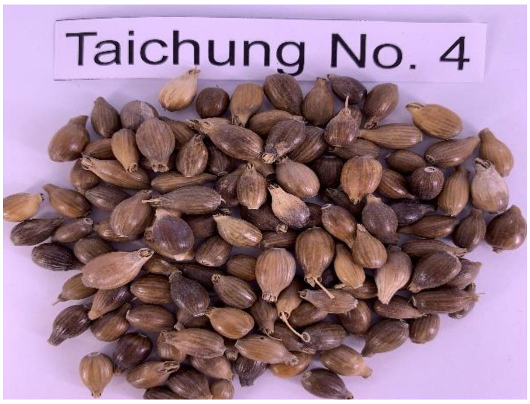

e) Taichung No. 5

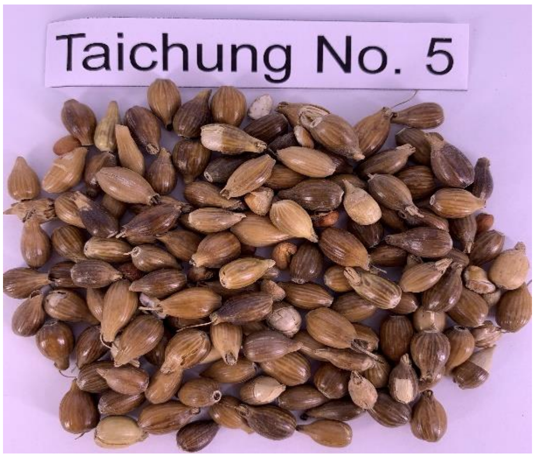

Figure S4. Morphology of coicis seeds.
* a-e: dehulled seeds from different cultivars.
